# Trastuzumab Emtansine Plus Non-Pegylated Liposomal Doxorubicin in HER2-Positive Metastatic Breast Cancer (Thelma): A Single-Arm, Multicenter, Phase Ib Trial

**DOI:** 10.3390/cancers12123509

**Published:** 2020-11-25

**Authors:** Elena López-Miranda, José Manuel Pérez-García, Serena Di Cosimo, Etienne Brain, Maja Ravnik, Santiago Escrivá-de-Romaní, Maria Vidal, Joseph Gligorov, Simona Borštnar, Laura Calabuig, Miguel Sampayo-Cordero, Andrea Malfettone, Antonio Llombart-Cussac, Thomas M. Suter, Javier Cortés

**Affiliations:** 1 Department of Medical Oncology, Hospital Universitario Ramón y Cajal, 28034 Madrid, Spain; elena.lopez@medsir.org; 2 Medical Department, Medica Scientia Innovation Research (MedSIR), Ridgewood, NJ 07450, USA; jose.perez@medsir.org (J.M.P.-G.); Serena.DiCosimo@istitutotumori.mi.it (S.D.C.); laura.calabuig@medsir.org (L.C.); sampayo.mc@gmail.com (M.S.-C.); andrea.malfettone@medsir.org (A.M.); allombart1@yahoo.com (A.L.-C.); 3 Medical Department, Medica Scientia Innovation Research (MedSIR), 08018 Barcelona, Spain; 4 International Breast Cancer Center (IBCC), Quiron Group, Medical Oncology Department, 08022 Barcelona, Spain; 5 Biomarkers Unit, Department of Applied Research and Technological Development, Fondazione IRCCS Istituto Nazionale dei Tumori, 20100 Milano, Italy; 6 Department of Medical Oncology, Institut Curie, 92210 St. Cloud, France; etienne.brain@curie.fr; 7 Department of Oncology, University Medical Centre Maribor, 2000 Maribor, Slovenia; maja.ravnik@ukc-mb.si; 8 Department of Medical Oncology, Vall d’Hebron Institute of Oncology (VHIO), Vall d’Hebron University Hospital, 08035 Barcelona, Spain; sescriva@vhio.net; 9 Department of Medical Oncology, Hospital Clinic, Barcelona, Translational Genomics and Targeted Therapies in Solid Tumors, IDIBAPS, 08036 Barcelona, Spain; MJVIDAL@clinic.cat; 10 Centre Expert Cancers du Sein Hôpital Tenon, Institut Universitaire de Cancérologie AP-HP. Sorbonne Université, 75020 Paris, France; joseph.gligorov@aphp.fr; 11 Division of Medical Oncology, Institute of Oncology Ljubljana, 1000 Ljubljana, Slovenia; sborstnar@onko-i.si; 12 Hospital Arnau de Vilanova, Universidad Católica de Valencia “San Vicente Mártir”, 46015 Valencia, Spain; 13 Department of Cardiology, Inselspital, Bern University Hospital, University of Bern, 3010 Bern, Switzerland; 14 Department of Medical Oncology, Vall d’Hebron Institute of Oncology (VHIO), 08035 Barcelona, Spain

**Keywords:** trastuzumab emtansine, T-DM1, non-pegylated liposomal doxorubicin, HER2-positive, metastatic breast cancer

## Abstract

**Simple Summary:**

Considering the favorable overall safety profile of trastuzumab emtansine (T-DM1), the low expected rate of cardiotoxicity, and the synergistic effect of anthracyclines with Human Epidermal Growth Factor Receptor 2 (HER2)-targeting agents, it is hypothesized that T-DM1 may be safely combined with non-pegylated liposomal doxorubicin (NPLD). In the THELMA trial, the effect of adding NPLD to T-DM1 was evaluated with the aim of enhancing T-DM1 efficacy using an extensive cardiological assessment in trastuzumab- and taxane-pretreated patients with HER2-positive metastatic breast cancer. Despite an unlikely drug synergism, this combination was generally well tolerated without clinically relevant worsening of cardiac function. No relationship was identified between early predictors of heart failure and left ventricular ejection fraction changes. Thus, the combination of T-DM1 plus NPLD is safe, but this regimen does not seem to improve T-DM1 antitumor activity in this setting.

**Abstract:**

The paper assesses the dose-limiting toxicities and the maximum tolerated dose (MTD) of trastuzumab emtansine (T-DM1) combined with non-pegylated liposomal doxorubicin (NPLD) in HER2-positive (HER2+) metastatic breast cancer (MBC). This single-arm, open-label, phase Ib trial (NCT02562378) enrolled anthracycline-naïve HER2+ MBC patients who had progressed on trastuzumab and taxanes. Patients received a maximum of 6 cycles of NPLD intravenously (IV) at various dose levels (45, 50, and 60 mg/m^2^) in the “3 plus 3” dose-escalation part. During expansion, they received 60 mg/m^2^ of NPLD every 3 weeks (Q3W) plus standard doses of T-DM1. The MTD was T-DM1 3.6 mg/kg plus NPLD 60 mg/m^2^ administered IV Q3W. No clinically relevant worsening of cardiac function was observed. Among all evaluable patients, the overall response rate was 40.0% (95%CI, 16.3–67.7) with a median duration of response of 6.9 months (95%CI, 4.8–9.1). Clinical benefit rate was 66.7% (95%CI, 38.4–88.2) and median progression-free survival was 7.2 months (95%CI, 4.5–9.6). No significant influence of NPLD on T-DM1 pharmacokinetics was observed. The addition of NPLD to T-DM1 is feasible but does not seem to improve the antitumor efficacy of T-DM1 in HER2+ MBC patients.

## 1. Introduction

Breast cancer is a heterogeneous disease with multiple clinical presentations and tumor characteristics [1]. The Human Epidermal Growth Factor Receptor 2 (*HER2*) gene encodes a 185-kDa transmembrane protein with tyrosine kinase activity. Amplification of the *HER2* protooncogene occurs in approximately 15–20% of patients with breast cancer and is an independent predictor of disease recurrence and breast cancer-related mortality [2].

There have been great advancements in HER2-targeting therapies converting HER2+ metastatic breast cancer (MBC) into a highly treatable disease and extend survival in most patients [3,4,5]. Trastuzumab emtansine (T-DM1) is an antibody–drug conjugate that efficiently combines the antitumor effects of trastuzumab to the cytotoxic potential of derivative of maytansine 1 (DM1), which is a potent microtubule polymerization inhibitor. The randomized EMILIA phase III trial randomly assigned 991 patients with HER2+ MBC who had previously been treated with trastuzumab and a taxane to receive T-DM1 or capecitabine plus lapatinib [6]. The positive results of this study led to the approval of T-DM1 for patients with HER2+ MBC previously treated with trastuzumab and a taxane. The overall safety profile in HER2+ MBC showed that T-DM1 was well tolerated and the most commonly reported high-grade adverse events (AEs) were laboratory abnormalities, such as transaminase elevation and thrombocytopenia [6,7,8]. Cardiotoxicity in terms of reduction in left ventricular ejection fraction (LVEF) from baseline of at least 15% to less than 50% was reported in 0.8% to 1.7% of patients who were treated with T-DM1 in the metastatic setting [6,7,8].

Doxorubicin is an effective anthracycline that is commonly used for patients with breast cancer. Nevertheless, its free formulation is associated with cumulative, dose-dependent cardiac toxicity, which limits its clinical use to mainly advanced settings [9,10]. The combination therapy of doxorubicin and trastuzumab has been proven to be highly active for patients with HER2+ MBC, but it has been associated with an increased risk of cardiotoxicity [11]. Risk factors were later identified for trastuzumab-associated cardiotoxicity, which has helped to reduce its frequency, but this combination is not routinely used in clinical practice.

Current available measures of cardiotoxicity—defined by decreases in LVEF using multigated acquisition (MUGA) scans or echocardiography—may become apparent only when myocardial damage is fully established, exceeding its ability to compensate, and potentially too late for any meaningful alterations in clinical management [12]. There is some evidence to suggest that both strain, strain rate, and serum cardiac markers, such as B-type natriuretic peptide (BNP) and troponin I, offer a more sensitive approach than LVEF to detecting subclinical cardiotoxicity at the earliest stages in patients with chronic heart failure [13,14,15]. Additionally, serum HER2 levels seem to be increased in patients who experienced chronic heart failure [16].

More recently, liposomal anthracycline formulations have been developed with the aim of increasing the therapeutic index of conventional anthracyclines. These liposomal formulations include non-pegylated liposomal doxorubicin (NPLD) and pegylated liposomal doxorubicin. Generally, these agents have been demonstrated to reduce cardiac toxicity significantly while maintaining similar antitumor efficacy compared to conventional anthracyclines [17,18,19]. In addition, the combination of liposomal anthracyclines with trastuzumab has been shown to be a safe and feasible treatment with promising antitumor activity in patients with treatment-naïve HER2+ MBC [20,21,22].

Considering the favorable overall safety profile of T-DM1, low expected rate of cardiotoxicity, and the synergistic effect of anthracyclines with HER2-targeting agents, it is hypothesized that T-DM1 may be safely combined with NPLD. This combination might offer a novel effective strategy for enhancing the antitumor effect of T-DM1. The aim of this phase Ib trial (NCT02562378) is to determine the dose-limiting toxicities (DLTs) and the maximum tolerated dose (MTD) of T-DM1 combined with NPLD in HER2+ MBC.

## 2. Results

### 2.1. Study Population

Between October 2015 and December 2017, a total of 15 patients with anthracycline-naïve HER2+, unresectable, locally advanced or MBC were enrolled at seven sites. Of these, 12 patients (80.0%) were distributed into three cohorts during the dose-escalation part (cohorts 1 and 2: three patients in each cohort; cohort 3: six patients), and three patients (20.0%) were included in the dose-expansion part. The median age was 50 years (range, 31–62 years), 86.7% had Eastern Cooperative Oncology Group (ECOG) performance status 0, 73.3% had estrogen-receptor positive tumors, 60.0% presented with “de novo” metastatic disease, and 73.3% had visceral disease (40.0% with liver metastases). A total of 11 (73.3%), 3 (20.0%), and 1 (6.7%) patients had received prior treatment for advanced disease in the first-line, second-line, and third-line setting, respectively.

All patients had previously been treated with a taxane and trastuzumab, and 80.0% had also previously received pertuzumab. Table 1 summarizes the patients’ baseline characteristics.

### 2.2. Treatment Exposure

A total of 11 patients (73.3%) completed six cycles of T-DM1 and NPLD: two patients in cohort 1, two patients in cohort 2, four patients in cohort 3, and three patients in the dose-expansion part. The median relative dose intensity for T-DM1 and NPLD was 90.6% and 85.9%, and the median duration of treatment was 6.3 and 3.7 months, respectively. At the time of the analysis (December 2018), all 15 patients had discontinued study treatment, most commonly because of disease progression (80.0%). Additional reasons for treatment discontinuation were AEs (6.7%), patient request (6.7%), and investigator decision (6.7%).

### 2.3. MTD Determination

No patient in cohorts 1 and 2 (45 and 50 mg/m^2^ NPLD dose levels, respectively) developed a DLT. One patient in cohort 3 (60 mg/m^2^ NPLD dose level) experienced a DLT consisting of grade 4 neutropenia lasting 13 days. This cohort was expanded to include three additional patients to confirm the safety and tolerability of the MTD with no other DLTs. Consequently, the MTD was determined to be 3.6 mg/kg of T-DM1 and 60 mg/m^2^ of NPLD IV on day 1 of each three-week cycle. The complete list of DLTs is provided in Table S1**.**

### 2.4. General Safety

All 15 patients received at least one dose of study treatment and were included in the safety analysis. All patients experienced at least one AE (grades 1–4). The most common treatment-related toxicities were neutropenia (*n* = 11, 73.3%), thrombocytopenia (*n* = 9, 60.0%), asthenia (*n* = 9, 60.0%), nausea (*n* = 9, 60.0%), elevation of liver transaminases (*n* = 8, 53.3%), decreased appetite (*n* = 5, 33.3%), and anemia (*n* = 4, 26.7%). These AEs were generally mild (grade 1/2) and reversible. Treatment-related AEs of any grade reported in ≥10% of patients are listed in Table 2.

Grade ≥3 treatment-related AEs occurred in nine patients (60.0%), and neutropenia was the most frequent (*n* = 8, 53.3%), but there were no instances of febrile neutropenia. Other grade ≥3 treatment-related AEs that occurred in ≥10% of patients included thrombocytopenia (*n* = 2, 13.3%) and elevation of liver transaminases (*n* = 2, 13.3%). One patient developed a hepatobiliary disorder (venoocclusive liver disease) that was not clearly related to the study drugs and led to treatment discontinuation. No grade 5 AEs or other unexpected safety issues were observed. Treatment-related AEs of grade ≥ 3 are summarized in Table 3.

### 2.5. Cardiac Safety

The median LVEF values at baseline were 64.1% (range, 59.3–71.0%), 67.0% (range, 60.0–72.0%), and 62.7% (range, 60.0–71.9%) in cohorts 1, 2, and 3, respectively. At the end of cycle 6, the median changes in LVEF values were 11.6% (range, 9.8–13.4%), −4.0% (range, −22.0–4.0%), and 0% (range, −5.0–5.0%), respectively. A summary of LVEF values is provided in Table S2.

No cases of LVEF decline to <50.0% or symptomatic heart failure were observed. There was an increase of cardiac markers (serum troponin I and BNP) during the study treatment with respect to the baseline, although the elevations were not clinically significant. Overall, 13 patients (86.7%) had at least one marker level above the upper limit of normal (ULN), and both levels were above the ULN in three patients (20.0%). Analyses of serum HER2 extracellular domain (ECD) levels did not reveal a relationship with either LVEF changes or cardiac markers elevation.

### 2.6. Antitumor Efficacy

With a median follow-up time of 9.8 months (range, 2.3–24.4 months), objective partial responses (PRs) were observed in six of 15 patients (40.0%). No patient attained complete response (CR). Overall response rate (ORR) was 33.3% (95%confidence intervals (CI), 0.8–90.6) in cohort 1, 66.7% (95%CI, 9.4–99.2) in cohort 2, and 33.3% (95%CI, 7.5–70.1) in cohort 3 as per Response Evaluation Criteria In Solid Tumors version 1.1 (RECIST v.1.1). A total of four patients had stable disease for 24 weeks or longer and of them, one patient in cohort 1 and three patients in cohort 3, leading to a clinical benefit rate (CBR) of 66.7% (95%CI, 38.4–88.2). Among responders the median duration of response (DoR) was 6.9 months (95%CI, 4.8–9.1). Of a total of 15 patients, only one patient (6.7%) in cohort 1 experienced progressive disease as the best response.

Of 11 patients in cohorts 2 and 3 who had received one prior treatment for advanced disease, five (45.5%) had PR and three (27.3%) had stable disease for 24 weeks or longer, with a CBR of 72.7% (95%CI, 39–94). Among responders with one prior line of treatment, the median DoR was 8.3 months (95%CI, 5.9–10.7) and median progression-free survival (PFS) was 7.2 months (95%CI, 6.6–7.8).

Median PFS was 8.2 months (95%CI, 1.3–10.3) in cohort 1, 7.0 months (95%CI, 3.8–not evaluable) in cohort 2, 7.2 months (95%CI, 4.5–9.6) in cohort 3, and 7.2 months in the overall study population (95%CI, 4.5–9.6).

**Figure 1:**
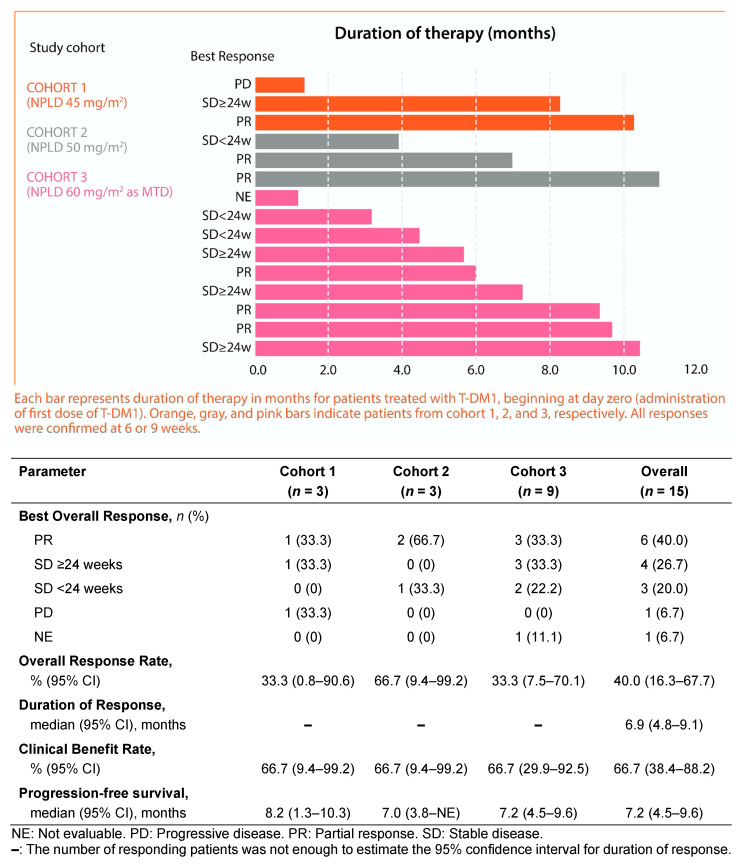
Antitumor clinical activity of the combination of T-DM1 and NPLD.

A summary of the antitumor clinical activity of the study treatment based on investigator review is provided in Figure 1.

### 2.7. Pharmacokinetics Analysis

Three subjects for each treatment cohort were included in the pharmacokinetic (PK) population. The PK parameters of the main substances (T-DM1 and doxorubicin) indicated that the mean plasma concentrations declined quickly in an exponential manner after the first infusion of the study treatment at each dose level. The mean PK parameters included the maximum concentration of drug observed in plasma (C_max_) and time from time zero to infinity (AUC_inf_) for T-DM1 were similar for each dose level of NPLD after the first administration of the study treatment with low inter-subject variability (coefficient of variation (CV) 4.0–30.0%). The mean C_max_ ranged from 67.8 to 79.6 μg/mL and AUC_inf_ ranged from 321 to 380 μg × day/mL. The median time required to reach the maximum concentration of drug in plasma (T_max_), mean time taken by the plasma concentration to reduce to 50% during the elimination phase (T_1/2_), body clearance (CL), and volume of distribution (V_d_) of T-DM1 were similar for each treatment cohort.

The mean total serum exposures of trastuzumab were approximately 1.6 to 2.3 times higher for cohort 2 than the exposures for the other cohorts. The mean total plasma exposures to DM1 ranged between 10.1 and 23.1 ng × d/mL among cohorts. Table 4, Table S3, and Table S4 summarize the PK results for T-DM1, total trastuzumab, and DM1.

Observations of the doxorubicin concentration–time curves were limited as concentrations fell below the quantification level at the collection point of 72 h post-infusion. The last measurable concentration time using the mean linear trapezoidal method (AUC_last_) ranged between 2.14 and 19.9 μg × h/mL, and the mean C_max_ was between 0.957 and 4.23 μg/mL with moderate to high inter-subject variability ranging from 48% and 127%. The median T_max_ values were similar for each treatment cohort.

In cycle 2, the results for mean plasma concentration versus time were similar to those observed in cycle 1 (Table 4). The PK parameters of the metabolite doxorubicinol are provided in the Table S5. The potential association between T-DM1 (or its unconjugated components), systemic exposure (C_max_ and AUC_inf_), and antitumor efficacy (ORR, CBR, and PFS) was analyzed by logistic regression. Neither statistically significant associations nor clear positive or negative trends were observed.

## 3. Discussion

In the metastatic setting, T-DM1 is approved as a single-agent to treat patients with HER2+, unresectable, locally advanced, or MBC who previously received trastuzumab and a taxane, either separately or in combination. Although T-DM1 has shown significant antitumor activity, less than half of the patients achieve an objective response, and all the patients eventually progress and require a new line of treatment [6].

Over the past few years, T-DM1 in combination with other agents has been explored because of its manageable safety profile, which makes it an ideal for combination treatment. Potential chemotherapy combinations have long been explored to improve T-DM1 efficacy in a metastatic setting but with negative results. Although the efficacy of the combination of T-DM1 plus docetaxel (with or without pertuzumab) was encouraging, this regimen was associated with significant toxicity, leading to dose reductions in nearly half of study patients [23]. Another study demonstrated that the addition of capecitabine to T-DM1 did not significantly improve patient outcome [24].

In this phase Ib study, the selected doses of T-DM1 and NPLD were 3.6 mg/kg and 60 mg/m^2^ every three weeks, respectively. These doses are the same as the recommended doses of either drug given alone. Moreover, based on comparison with historical controls, no PK interaction was observed between NPLD and T-DM1, and T-DM1 PKs have been consistent with those reported for T-DM1 given as a monotherapy [25].

Unfortunately, the addition of NPLD does not appear to increase the antitumor activity of T-DM1 significantly as a single agent, with a median PFS of 7.2 months, an ORR of 40.0%, and a CBR of 66.7% in a trastuzumab- and taxane-pretreated population (refer to the Tables S6 and S7 for a comprehensive overview of efficacy of T-DM1-based and NPLD-based regimens in landmark trials, respectively). These findings do not significantly differ from those achieved with T-DM1 in the EMILIA trial (median PFS of 9.6 months and ORR of 43.6%) [6]. Nevertheless, in contrast to the EMILIA trial, most of the patients included in this study previously received pertuzumab, which has been associated with reduced T-DM1 efficacy [25]. This fact, along with the limited number of patients, does not allow us to draw definite conclusions.

The safety profiles of T-DM1 and NPLD were consistent with previous reports, with no new safety findings for either agent, and AEs were generally manageable (refer to the Tables S6 and S8 for a comprehensive overview of the safety of T-DM1-/NPLD-containing regimens in landmark trials and THELMA). Myelosuppression was the most frequent toxicity, but the addition of NPLD did not significantly increase the incidence of severe thrombocytopenia typically associated with T-DM1. However, hepatotoxicity was slightly higher with the combination of NPLD and T-DM1 than previously reported with T-DM1 as a single-agent, and one patient discontinued the study treatment due to venoocclusive liver disease, although it was probably not related to study drugs [6].

The addition of NPLD was not associated with significant cardiotoxicity, and no patients developed asymptomatic LVEF declines or symptomatic heart failure. However, some patients presented an elevation of cardiac markers (troponin I and BNP) that was not clinically significant during the study treatment. It is important to emphasize that prior treatment with anthracyclines was not allowed, and this patient selection could have helped to obtain this favorable cardiac safety profile. No relevant correlation with cardiotoxicity was observed in the analysis of serum HER2 ECD levels.

## 4. Materials and Methods

### 4.1. Patient Population

Eligible patients were age ≥18 years with HER2+ (according to 2013 American Society of Clinical Oncology/College of American Pathologists (ASCO/CAP) guidelines), unresectable, locally advanced or MBC by local testing. Patients could have previously received up to two prior chemotherapy regimens in an advanced setting, and the disease had to have progressed or relapsed during or after taxane—and trastuzumab—based therapy. Other inclusion criteria were ECOG performance status ≤1 and adequate bone marrow, renal, hepatic, and cardiovascular function (LVEF ≥55%). Patients were required to have evaluable or measurable disease according to RECIST v.1.1.

The major exclusion criteria included previous treatment with T-DM1 or anthracyclines, either in a (neo)adjuvant or metastatic setting; central nervous system involvement (except if the patient was >4 weeks from radiotherapy completion, clinically stable, and not receiving steroids at study entry); cardiopulmonary dysfunction; history of a LVEF decline to <40.0% or symptomatic heart failure with previous trastuzumab-based treatment; and peripheral neuropathy of grade 3 or higher according to the National Cancer Institute (NCI) Common Terminology Criteria for Adverse Events (CTCAE) version 4.0. This study was performed in agreement with the guidelines of the International Conference on Harmonization, the ethical principles of the Declaration of Helsinki, and all applicable regulations. Informed consent was obtained from all participants. This study was approved by regulatory authorities and the following ethics committees: the Comité Ético de Investigación Clínica of the Vall d’Hebron University Hospital in Barcelona, Spain, on January 9, 2015 with code ID-RTF020; the Comité de Protection des Personnes, Hôpital Saint-Antoine in Paris, France on February 3, 2015, with code 14994; and Komisija za Medicinsko Etiko, Ministrstvo za Zdravje, Ljubljana, Slovenia on September 27, 2017, with code 0120-425/2017-8.

### 4.2. Study Design

THELMA (NCT02562378) was a single-arm, open-label, multicenter, phase Ib trial of T-DM1 in combination with NPLD. The trial employed a standard “3 plus 3” dose-escalation design with a dose-expansion cohort at the MTD. Patients were treated with 3.6 mg/kg of T-DM1 intravenously (IV) on day 1 of each three-week cycle and increasing doses of NPLD during the dose-escalation part (45 mg/m^2^, 50 mg/m^2^, and 60 mg/m^2^ IV on day 1 of each cycle for up to 6 cycles). A de-escalation part was planned with 3.6 mg/kg IV of T-DM1 and 15 mg/m^2^ IV of NPLD weekly if more than one of six (1/6) patients included in the first dose period experienced a DLT. After cycle 6, patients could continue with T-DM1 as a single-agent until disease progression, the development of unacceptable toxicity, investigator decision, or withdrawal of consent.

The primary endpoint was to establish the MTD, which was defined as the highest dose level at which either one of six (1/6) or none of three (0/3) patients experienced a DLT during the first two cycles of study treatment. DLT was defined as the occurrence of any of the treatment-related AEs listed in Table S1 during the first two cycles of study treatment. The population for primary analysis included those patients who completed the first two cycles or stopped study treatment during that time because of DLT. Toxicities were defined using NCI CTCAE version 4.0.

Preliminary efficacy was assessed by the ORR, CBR, DoR, and PFS. Additional secondary objectives included both the general and cardiac safety profiles of T-DM1 plus NPLD, the evaluation of serum HER2 ECD levels as a predictor of cardiac toxicity, and the assessment of PKs interactions between NPLD and T-DM1. The intention-to-treat (ITT) population included every patient who received any dose of study treatment and entered the safety analysis. Patients with a complete treatment concentration time profile constituted the population for PK analysis.

### 4.3. Study Assessments

Physical examination, ECOG status, and laboratory assessments were conducted at baseline and on day 1 of each three-week cycle. Cardiac assessments (echocardiogram, 12-lead electrocardiogram (ECG), and cardiac markers serum troponin I and BNP) were performed at baseline and on day 1 of each cycle during treatment with T-DM1 and NPLD (cycles 1–6). In addition, cardiac markers were also determined on days 8 and 15 during the first two cycles of study treatment. Thereafter, echocardiograms and ECG were performed every 9 weeks until the end of study (EoS) visit.

PK samples for T-DM1, trastuzumab, and DM1 were collected at predetermined time points in cycles 1, 2, and 4. PK samples for doxorubicin and doxorubicinol were obtained only in cycles 1 and 2. Tumor assessment was performed at the end of cycles 2, 4, and 6. Afterwards, tumor assessment was conducted every 9 weeks until the EoS visit. Response assessment was evaluated by the investigator based on physical examinations, computerized tomography, or magnetic resonance imaging scans, and bone scintigraphy using RECIST v.1.1.

ORR was defined as the proportion of patients with the best overall response (either CR or PR) according to RECIST v.1.1 and confirmed at least 4 weeks after the initial response. CBR was defined as the proportion of patients with the best overall response (either CR or PR) or stable disease lasting 24 weeks or longer. PFS was defined as the time from the initiation of study treatment to disease progression or death from any cause.

### 4.4. Serum HER2 ECD Assessment

Peripheral blood was collected at baseline and on cycle 4 in a sterile test tube and following centrifugation serum samples were stored at −20 °C until the time of the assay. After collection of all samples, serum HER2 ECD concentrations were determined by Enzyme-Linked Immunosorbent Assay (ELISA) using the ADVIA^®^ Centaur XP Immunoassay System (Siemens Diagnostics^®,^ Tarrytown, NY, USA) with a detection range of 0.5–350 ng/mL. The assay was conducted in accordance with the manufacturers’ instructions and blinded to both patients’ characteristics and clinical outcomes.

### 4.5. PK Evaluation

PK analysis was performed with the data of all subjects using the software Phoenix WinNonlin^®^ version 8.0 (Pharsight, St. Louis, MO, USA) and SAS version 9.3 (SAS Institute, Cary, NC, USA). All concentration values that were below the limit of quantification were considered as zero. Missing values were not included in the PK analysis. The estimated C_max_, T_max_, T_1/2_, and the elimination rate constant (Lambda_z). The area under the curve of plasma concentration versus time was also calculated from time zero to the AUC_last_.

The area under the curve of concentration versus AUC_inf_ was calculated using the linear trapezoidal rule, and the extrapolated AUC percentage of total AUC was calculated as

[AUC_inf_ − AUC_last_/AUC_inf_] × 100 (AUC_ext_) (1)

Total CL was calculated as the total dose (mg) divided by AUC_inf_ (CL), and the V_d_ based on the terminal was calculated as

[CL/Lambda_z](2)

When AUC_ext_ was greater than 20%, AUC_inf_ and its associated parameters (T_1/2_, CL, and V_d_) were set as missing, and AUC_last_ was reported. A non-compartmental method (Model 200 of Phoenix WinNonlin^®^ 5.2, Pharsight, St. Louis, MO, USA) was used to estimate the PK parameters of T-DM1, total trastuzumab, and DM1.

### 4.6. Statistical Analysis

The target sample size was established as ranging between 12 and 24 patients depending on the number of cohorts required to determine the MTD. The secondary binary efficacy endpoints (ORR and CBR) were described with percentages and 95% Clopper–Pearson CI. For the time-to-event endpoint (PFS and DoR), the median and 95% CIs were reported based on the Kaplan–Meier method. DoR was reported with waterfall plots. Exploratory objectives were analyzed using descriptive statistics.

PK parameters for T-DM1 and total trastuzumab in serum, DM1 in plasma, and doxorubicin and doxorubicinol in plasma were summarized using descriptive statistics (number of patients (N), mean, standard deviation (SD), median, CV, minimum, and maximum). The exposure–response relationship for ORR, CBR, and PFS was analyzed by logistic regression analysis. P-values and 95% CIs were based on the likelihood ratio and profile-likelihood method. The level of significance was set at 5%.

## 5. Conclusions

In conclusion, the combination of NPLD and T-DM1 is feasible, but the addition of NPLD does not seem to enhance the antitumor efficacy of T-DM1 in patients with HER2+ MBC. These findings do not support further development of this combination. However, despite these and more disappointing results with other combinations, optimization of T-DM1 therapy with other agents remains an unmet need for HER2+ MBC patients.

## Figures and Tables

**Table 1:** Demographic and baseline patient characteristics.

Characteristic	Cohort 1 (*n* = 3)	Cohort 2 (*n* = 3)	Cohort 3 (*n* = 9)	Overall (*n* = 15)
**Age, median (range), years**				
	50.0 (39.0–62.0)	58.0 (57.0–61.0)	42.0 (31.0–62.0)	50.0 (31.0–62.0)
**ECOG performance status, *n* (%)**				
0	3 (100)	3 (100)	7 (77.8)	13 (86.7)
1	0 (0)	0 (0)	2 (22.2)	2 (13.3)
HER2 expression, *n* (%)				
IHC 3+	2 (66.7)	2 (66.7)	7 (77.8)	11 (73.3)
IHC 2+ and ISH+	1 (33.3)	1 (33.3)	2 (22.2)	4 (26.7)
**Hormone receptor status, *n* (%)**				
ER-positive	2 (66.7)	3 (100)	6 (66.7)	11 (73.3)
ER-negative	1 (33.3)	0 (0)	3 (33.3)	4 (26.7)
PR-positive	1 (33.3)	2 (66.7)	4 (44.4)	7 (46.7)
PR-negative	2 (66.7)	1 (33.3)	5 (55.6)	8 (53.3)
**Disease stage at initial diagnosis, *n* (%)**				
I	0 (0)	0 (0)	1 (11.1)	1 (6.7)
II	0 (0)	0 (0)	2 (22.2)	2 (13.3)
III	0 (0)	0 (0)	3 (33.3)	3 (20.0)
IV	3 (100)	3 (100)	3 (33.3)	9 (60.0)
**De novo metastatic disease, *n* (%)**				
Yes	3 (100)	3 (100)	3 (33.3)	9 (60)
No	0 (0)	0 (0)	6 (66.7)	6 (40)
**Sites of metastases, *n* (%)**				
Lymph Node	2 (66.7)	1 (33.3)	7 (77.8)	10 (66.7)
Bone	3 (100)	3 (100)	4 (44.4)	10 (66.7)
Liver	0 (0)	2 (66.7)	4 (44.4)	6 (40.0)
Lung	0 (0)	1 (33.3)	4 (44.4)	5 (33.3)
Brain	1 (33.3)	0 (0)	1 (11.1)	2 (13.3)
Skin	0 (0)	0 (0)	2 (22.2)	2 (13.3)
Others	0 (0)	1 (33.3)	1 (11.1)	2 (13.3)
**Lines of previous treatment for advanced disease, *n* (%)**				
1	0 (0)	3 (100)	8 (88.9)	11 (73.3)
2	3 (100)	0 (0)	0 (0)	3 (20.0)
3	0 (0)	0 (0)	1 (11.1)	1 (6.7)
**Prior taxane treatment, *n* (%)**				
	3 (100)	3 (100)	9 (100)	15 (100)
**Prior anthracycline treatment, *n* (%)**				
	0 (0)	0 (0)	0 (0)	0 (0)
**Prior trastuzumab treatment, *n* (%)**				
	3 (100)	3 (100)	9 (100)	15 (100)
**Prior pertuzumab treatment, *n* (%)**				
	1 (33.3)	3 (100)	8 (88.9)	12 (80.0)

**Table 2:** Treatment-related adverse events of any grade occurring in more than 10% of patients.

Adverse Event	Cohort 1 (*n* = 3) *n* (%)	Cohort 2 (*n* = 3) *n* (%)	Cohort 3 (*n* = 9) *n* (%)	Overall (*n* = 15) *n* (%)
**Hematological**				
Neutropenia	1 (33.3)	3 (100)	7 (77.8)	11 (73.3)
Thrombopenia	1 (33.3)	2 (66.7)	6 (66.7)	9 (60.0)
Anemia	1 (33.3)	1 (33.3)	4 (44.4)	4 (26.7)
Leukopenia	0 (0)	1 (33.3)	2 (22.2)	3 (20.0)
Lymphopenia	0 (0)	1 (33.3)	2 (22.2)	3 (20.0)
Decreased hemoglobin	1 (33.3)	1 (33.3)	0 (0)	2 (13.3)
Decreased lymphocyte count	0 (0)	1 (33.3)	1 (11.1)	2 (13.3)
**Non-Hematological**				
Asthenia	3 (100)	2 (66.7)	4 (44.4)	9 (60.0)
Nausea	2 (66.7)	3 (100)	4 (44.4)	9 (60.0)
Increased aspartate aminotransferase	1 (33.3)	1 (33.3)	6 (66.7)	8 (53.3)
Increased alanine aminotransferase	1 (33.3)	1 (33.3)	4 (44.4)	6 (40.0)
Increased brain natriuretic peptide	0 (0)	2 (66.7)	4 (44.4)	6 (40.0)
Increased gamma-glutamyl transferase	2 (66.7)	2 (66.7)	2 (22.2)	6 (40.0)
Increased troponin I	0 (0)	1 (33.3)	4 (44.4)	5 (33.3)
Decreased appetite	1 (33.3)	1 (33.3)	3 (33.3)	5 (33.3)
Alopecia	0 (0)	1 (33.3)	3 (33.3)	4 (26.7)
Epistaxis	0 (0)	1 (33.3)	2 (22.2)	3 (20.0)
Rhinorrhea	0 (0)	1 (33.3)	2 (22.2)	3 (20.0)
Headache	0 (0)	2 (66.7)	0 (0)	2 (13.3)
Fatigue	0 (0)	0 (0)	2 (22.2)	2 (13.3)
Mucosal inflammation	0 (0)	0 (0)	2 (22.2)	2 (13.3)
Increased blood alkaline phosphatase	0 (0)	1 (33.3)	1 (11.1)	2 (13.3)
Aphthous ulcer	0 (0)	1 (33.3)	1 (11.1)	2 (13.3)
Constipation	1 (33.3)	0 (0)	1 (11.1)	2 (13.3)
Diarrhea	1 (33.3)	0 (0)	1 (11.1)	2 (13.3)
Dry mouth	0 (0)	1 (33.3)	1 (11.1)	2 (13.3)
Gingival bleeding	0 (0)	0 (0)	2 (22.2)	2 (13.3)
Vomiting	0 (0)	1 (33.3)	1 (11.1)	2 (13.3)
Hypoalbuminemia	0 (0)	1 (33.3)	1 (11.1)	2 (13.3)
Rash	0 (0)	1 (33.3)	1 (11.1)	2 (13.3)

**Table 3:** Grade 3–5 treatment-related adverse events occurring in the safety population.

Adverse event	Cohort 1 (*n* = 3)	Cohort 2 (*n* = 3)	Cohort 3 (*n* = 9)	Overall (*n* = 15)
**Hematological**				
Neutropenia	0 (0)	2 (66.7)	6 (66.7)	8 (53.3)
Thrombopenia	0 (0)	0 (0)	2 (22.2)	2 (13.3)
Leukopenia	0 (0)	1 (33.3)	1 (11.1)	2 (13.3)
Lymphopenia	0 (0)	1 (33.3)	1 (11.1)	2 (13.3)
**Non-Hematological**				
Increased aspartate aminotransferase	0 (0)	0 (0)	2 (22.2)	2 (13.3)
Fatigue	0 (0)	0 (0)	1 (11.1)	1 (6.7)

**Table 4:** Pharmacokinetic parameters of trastuzumab emtansine (T-DM1) and doxorubicin by treatment dose level.

Treatment Dose Level	T-DM1 3.6 mg/kg Plus NPLD 45 mg/m^2^ (*n* = 3)		T-DM1 3.6 mg/kg Plus NPLD 50 mg/m^2^ (*n* = 6)		T-DM1 3.6 mg/kg Plus NPLD 60 mg/m^2^ (*n* = 9)	
**CYCLE 1**						
**Parameter**	**T-DM1** **mean (% CV)**	**Doxorubicin** **mean (% CV)**	**T-DM1** **mean (% CV)**	**Doxorubicin** **mean (% CV)**	**T-DM1** **mean (% CV)**	**Doxorubicin** **mean (% CV)**
AUC_inf_ (μg × h/mL) ^a^	355 (15.2)	NE	380 (29.1)	NE	321 (18.6)	NE
AUC_last_ (μg × h/mL)	348 (14.4)	2.14 (89.6)	372 (30.3)	19.9 (126.6)	317 (18.9)	10.8 (68.3)
C_max_ (μg/mL)	73.3 (3.6)	0.957 (88.2)	79.6 (28.5)	4.23 (91.5)	67.8 (17.8)	2.68 (47.5)
T_max_ (h) ^b^	1.95 (1.83–2.00)	1.08 (1.08–1.17)	1.83 (1.83–2.02)	1.17 (1.08–1.43)	1.95 (1.80–2.08)	1.17 (1.13–1.33)
T_1/2_ (days) ^a^	3.57 (33.8)	NE	4.25 (13.2)	NE	3.48 (11.7)	NE
V_d_ (mL/kg) ^a^	50.1 (10.1)	NE	56.4 (19.5)	NE	55.9 (21.6)	NE
Cl (mL/kg/day) ^a^	10.0 (12.1)	NE	10.1 (32.1)	NE	11.0 (16.3)	NE
**CYCLE 2**						
**Parameter**	**T-DM1** **mean (% CV)**	**Doxorubicin** **mean (% CV)**	**T-DM1** **mean (% CV)**	**Doxorubicin** **mean (% CV)**	**T-DM1** **mean (% CV)**	**Doxorubicin** **mean (% CV)**
AUC_last_ (μg × h/mL)	NA	3.85 (45.6)	NA	21.5 (122.4)	NA	8.27 (42.7)
C_max_ (μg/mL)	NA	1.58 (52.6)	NA	4.71 (86.2)	NA	2.57 (28.3)
T_max_ (h) ^a^	NA	1.15 (1.10–1.35)	NA	1.17 (1.08–1.17)	NA	1.33 (1.13–1.37)

^a^ Lambda z-dependent parameter (time from time zero to infinity (AUC_inf_), mean time taken by the plasma concentration to reduce to 50% during the elimination phase (T_1/2_), body clearance (Cl), and volume of distribution (V_d_)) were not estimated for doxorubicin. ^b^ Median (minimum and maximum) are reported for the median time required to reach the maximum concentration of drug in plasma (T_max_). NE: Not estimated. NA: Not applicable.

## Supplementary Materials

The following are available online at https://www.mdpi.com/2072-6694/12/12/3509/s1. Table S1. List of dose-limiting toxicities (DLTs); Table S2. Evolution of median left ventricular ejection fraction (LVEF) values at baseline and cycle 6 in the three study cohorts; Table S3. Pharmacokinetic parameters for trastuzumab by treatment dose level; Table S4. Pharmacokinetic parameters for DM1 by treatment dose level; Table S5. Pharmacokinetic parameters for doxorubicinol by treatment dose level; Table S6. The efficacy and safety of T-DM1–containing regimens for HER2-positive breast cancer; Table S7. The efficacy of non-pegylated liposomal doxorubicin-based regimens for metastatic breast cancer; Table S8. The safety of non-pegylated liposomal doxorubicin-based regimens for metastatic breast cancer.

## References

[1] Perou C.M., Sørlie T., Eisen M.B., van de Rijn M., Jeffrey S.S., Rees C.A., Pollack J.R., Ross D.T., Johnsen H., Akslen L.A. (2000). Molecular portraits of human breast tumours. Nature.

[2] Hudis C.A. (2007). Trastuzumab--mechanism of action and use in clinical practice. N. Engl. J. Med..

[3] Cesca M.G., Vian L., Cristóvão-Ferreira S., Pondé N., de Azambuja E. (2020). HER2-positive advanced breast cancer treatment in 2020. Cancer Treat. Rev..

[4] Kunte S., Abraham J., Montero A.J. (2020). Novel HER2-targeted therapies for HER2-positive metastatic breast cancer. Cancer.

[5] Leo C.P., Hentschel B., Szucs T.D., Leo C. (2020). FDA and EMA Approvals of New Breast Cancer Drugs-A Comparative Regulatory Analysis. Cancers.

[6] Verma S., Miles D., Gianni L., Krop I.E., Welslau M., Baselga J., Pegram M., Oh D.-Y., Diéras V., Guardino E. (2012). Trastuzumab emtansine for HER2-positive advanced breast cancer. N. Engl. J. Med..

[7] Krop I.E., Kim S.-B., González-Martín A., LoRusso P.M., Ferrero J.-M., Smitt M., Yu R., Leung A.C.F., Wildiers H. (2014). TH3RESA study collaborators Trastuzumab emtansine versus treatment of physician’s choice for pretreated HER2-positive advanced breast cancer (TH3RESA): A randomised, open-label, phase 3 trial. Lancet Oncol..

[8] Perez E.A., Barrios C., Eiermann W., Toi M., Im Y.-H., Conte P., Martin M., Pienkowski T., Pivot X., Burris H.A. (2017). Trastuzumab Emtansine with or Without Pertuzumab Versus Trastuzumab Plus Taxane for Human Epidermal Growth Factor Receptor 2–Positive, Advanced Breast Cancer: Primary Results From the Phase III MARIANNE Study. J. Clin. Oncol..

[9] Singal P.K., Iliskovic N. (1998). Doxorubicin-induced cardiomyopathy. N. Engl. J. Med..

[10] Von Hoff D.D., Layard M.W., Basa P., Davis H.L., Von Hoff A.L., Rozencweig M., Muggia F.M. (1979). Risk factors for doxorubicin-induced congestive heart failure. Ann. Intern. Med..

[11] Slamon D.J., Leyland-Jones B., Shak S., Fuchs H., Paton V., Bajamonde A., Fleming T., Eiermann W., Wolter J., Pegram M. (2001). Use of Chemotherapy plus a Monoclonal Antibody against HER2 for Metastatic Breast Cancer That Overexpresses HER2. N. Engl. J. Med..

[12] Ewer M.S., Lenihan D.J. (2008). Left Ventricular Ejection Fraction and Cardiotoxicity: Is Our Ear Really to the Ground?. JCO.

[13] Zardavas D., Suter T.M., Van Veldhuisen D.J., Steinseifer J., Noe J., Lauer S., Al-Sakaff N., Piccart-Gebhart M.J., de Azambuja E. (2016). Role of Troponins I and T and N-Terminal Prohormone of Brain Natriuretic Peptide in Monitoring Cardiac Safety of Patients With Early-Stage Human Epidermal Growth Factor Receptor 2–Positive Breast Cancer Receiving Trastuzumab: A Herceptin Adjuvant Study Cardiac Marker Substudy. JCO.

[14] Perik P.J., de Vries E.G.E., Gietema J.A., van der Graaf W.T.A., Smilde T.D.J., Sleijfer D.T., Veldhuisen D.J. (2007). van Serum HER2 levels are increased in patients with chronic heart failure. Eur. J. Heart Fail..

[15] Sawaya H., Sebag I.A., Plana J.C., Januzzi J.L., Ky B., Cohen V., Gosavi S., Carver J.R., Wiegers S.E., Martin R.P. (2011). Early detection and prediction of cardiotoxicity in chemotherapy-treated patients. Am. J. Cardiol..

[16] Chan S., Davidson N., Juozaityte E., Erdkamp F., Pluzanska A., Azarnia N., Lee L.W. (2004). Phase III trial of liposomal doxorubicin and cyclophosphamide compared with epirubicin and cyclophosphamide as first-line therapy for metastatic breast cancer. Ann. Oncol..

[17] Batist G., Ramakrishnan G., Rao C.S., Chandrasekharan A., Gutheil J., Guthrie T., Shah P., Khojasteh A., Nair M.K., Hoelzer K. (2001). Reduced cardiotoxicity and preserved antitumor efficacy of liposome-encapsulated doxorubicin and cyclophosphamide compared with conventional doxorubicin and cyclophosphamide in a randomized, multicenter trial of metastatic breast cancer. J. Clin. Oncol..

[18] Harris L., Batist G., Belt R., Rovira D., Navari R., Azarnia N., Welles L., Winer E. (2002). TLC D-99 Study Group Liposome-encapsulated doxorubicin compared with conventional doxorubicin in a randomized multicenter trial as first-line therapy of metastatic breast carcinoma. Cancer.

[19] Chia S., Clemons M., Martin L.-A., Rodgers A., Gelmon K., Pond G.R., Panasci L. (2006). Pegylated liposomal doxorubicin and trastuzumab in HER-2 overexpressing metastatic breast cancer: A multicenter phase II trial. J. Clin. Oncol..

[20] Cortes J., Cosimo S.D., Climent M.A., Cortés-Funes H., Lluch A., Gascón P., Mayordomo J.I., Gil M., Benavides M., Cirera L. (2009). Nonpegylated Liposomal Doxorubicin (TLC-D99), Paclitaxel, and Trastuzumab in HER-2-Overexpressing Breast Cancer: A Multicenter Phase I/II Study. Clin. Cancer Res..

[21] Baselga J., Manikhas A., Cortés J., Llombart A., Roman L., Semiglazov V.F., Byakhov M., Lokanatha D., Forenza S., Goldfarb R.H. (2014). Phase III trial of nonpegylated liposomal doxorubicin in combination with trastuzumab and paclitaxel in HER2-positive metastatic breast cancer. Ann. Oncol..

[22] Martin M., Fumoleau P., Dewar J.A., Albanell J., Limentani S.A., Campone M., Chang J.C., Patre M., Strasak A., de Haas S.L. (2016). Trastuzumab emtansine (T-DM1) plus docetaxel with or without pertuzumab in patients with HER2-positive locally advanced or metastatic breast cancer: Results from a phase Ib/IIa study. Ann. Oncol..

[23] Cortes J., Dieras V., Lorenzen S., Montemurro F., Riera-Knorrenschild J., Thuss-Patience P., Allegrini G., Laurentiis M.D., Lichinitser M., Lohrisch C. (2018). Abstract CT096: Trastuzumab emtansine (T-DM1) + capecitabine in HER2-positive metastatic breast cancer (mBC) and HER2-positive locally advanced (LA)/metastatic gastric cancer (mGC): Results from the phase I/randomized phase II TRAXHER2 study. Cancer Res..

[24] Krop I.E., Beeram M., Modi S., Jones S.F., Holden S.N., Yu W., Girish S., Tibbitts J., Yi J.-H., Sliwkowski M.X. (2010). Phase I study of trastuzumab-DM1, an HER2 antibody-drug conjugate, given every 3 weeks to patients with HER2-positive metastatic breast cancer. J. Clin. Oncol..

[25] Dzimitrowicz H., Berger M., Vargo C., Hood A., Abdelghany O., Raghavendra A.S., Tripathy D., Valero V., Hatzis C., Pusztai L. (2016). T-DM1 Activity in Metastatic Human Epidermal Growth Factor Receptor 2–Positive Breast Cancers That Received Prior Therapy With Trastuzumab and Pertuzumab. JCO.

