# Supplementary material for: Trastuzumab Emtansine Plus Non-Pegylated Liposomal Doxorubicin in HER2-Positive Metastatic Breast Cancer (Thelma): A Single-Arm, Multicenter, Phase Ib Trial
Source: Cancers (Basel). 2020 Nov 25;12(12):3509. doi: 10.3390/cancers12123509 (PMC7760511; doi:10.3390/cancers12123509)
Supplement: Supplementary file 1 [file cancers-12-03509-s001.pdf]

cancers

MDPI

Article

Trastuzumab Emtansine Plus Non-Pegylated Liposomal Doxorubicin in HER2-Positive Metastatic Breast Cancer (Thelma): A Single-Arm, Multicenter, Phase Ib Trial

Elena López-Miranda, José Manuel Pérez-García, Serena Di Cosimo, Etienne Brain, Maja Ravnik, Santiago Escrivá-de-Romaní, Maria Vidal, Joseph Gligorov, Simona Borštnar, Laura Calabuig, Miguel Sampayo-Cordero, Andrea Malfettone, Antonio Llombart-Cussac, Thomas M. Suter and Javier Cortés

Table 1. List of dose-limiting toxicities (DLTs).

Event	Type of DLT
**Hematological toxicity**	- Grade 4 neutropenia (i.e. absolute neutrophil count (ANC) < 0.5 x 10^9 cells/L for minimal duration of 7 days).<br>- Grades 3 and 4 febrile neutropenia (i.e. ANC < 1.0 x 10^9 cells/L with a single temperature of >38.3°C or a sustained temperature of ≥ 38°C for more than one hour).<br>- Uncomplicated Grade 4 thrombocytopenia (< 25.0 x 10^9 cells/L) which does not recover to ≥ 75.0 x 10^9 cells/L before next planned dose administration.<br>- Thrombocytopenia (any grade) complicated with clinically significant bleeding requiring medical intervention, such as platelet transfusion or cauterization [a].
**Cardiac toxicity**	Level I cardiotoxicity defined as:<br>- Sudden death (defined as within 24 hours; unexplained)<br>- Heart failure NYHA criteria class III-IV and LVEF decline defined as an absolute drop ≥10% resulting in a final LVEF <50%
**Hepatic toxicity**	- Increase in AST (SGOT)/ALT (SGPT) values to >5x ULN<br>- Increase in total bilirubin value to > 3xULN<br>- Abnormalities meeting the Hy's Law [b].
**Others**	- Grade ≥ 3 non-hematological toxicities [c].<br>- Any treatment-related non-hematological toxicity grade ≥ 3 preventing the start of the 3rd cycle on Day 42 (6 weeks cycle length)<br>- Grade 2 non-hematological toxicity requiring interruption of treatment for > 21 days<br>- Patient not able to receive 100% of the dose level going into Cycle 3, Day 1

[a] Patients with Grade 1 or 2 epistaxis may have cauterization, and this should not be considered as a DLT.[b] Defined by The U.S. Department of Health and Human Services, Food and Drug Administration, Center for Drug Evaluation and Research (CDER) Center for Biologics Evaluation and Research (CBER) as the rule of thumb that a drug is at high risk of causing a fatal drug-induced liver injury (DILI) when given to a large population, if it caused cases of liver injury that satisfied certain criteria when given to a smaller population. Hy's Law cases have the following three components: a) the drug causes hepatocellular injury, generally shown by more frequent 3-fold or greater elevations above the upper limits of normal (ULN) of ALT or AST than the (nonhepatotoxic) control agent or placebo; b) among subjects showing such aminotransferase (AT) elevations, often with ATs much greater than 3xULN, some subjects also show elevation of serum total bilirubin (TBL) to >2xULN, without initial findings of cholestasis (serum alkaline phosphatase (ALP) activity

>2xULN); c) no other reason can be found to explain the combination of increased AT and serum TBL, such as viral hepatitis A, B, or C, pre-existing or acute liver disease, or another drug capable of causing the observed injury. [c] Excepting: a) grade ≥ 3 diarrhea that recovers to grade ≤ 2 after 24 hours of starting recommended antidiarrheal treatment, b) grade 3 nausea, vomiting or diarrhea without appropriate treatment, c) grade 3 or 4 nausea or anorexia that resolves to grade 1 prior to the start next cycle, d) infusion-related reactions (although precautions will be taken if IRR grade ≥ 3 occur), and e) laboratory values of ≥ grade 3 which are judged not clinically significant by the investigator.

Table 2. Evolution of median left ventricular ejection fraction (LVEF) values at baseline and cycle 6 in the three study cohorts.

LVEF (%)	T-DM1 3.6 mg/kg plus NPLD 45 mg/m² (*n* = 3)	T-DM1 3.6 mg/kg plus NPLD 50 mg/m² (*n* = 3)	T-DM1 3.6 mg/kg plus NPLD 60 mg/m² (*n* = 3)
**Baseline**			
N valid	3	3	9
Mean (SD)	64.8 (5.9)	66.3 (6.0)	63.4 (4.2)
Median (Min, Max)	64.1 (59.3–71.0)	67.0 (60.0–72.0)	62.7 (60.0–71.9)
Overall assessment			
Normal, *n* (%)	3 (100)	3 (100)	9 (100)
**Change from Baseline to Cycle 6 Day 21**			
N valid	2	3	6
Mean (SD)	11.6 (2.5)	-7.3 (13.3)	0.1 (3.9)
Median (Min, Max)	11.6 (9.8–13.4)	-4.0 (-22.0–4.0)	0.0 (-5.0–5.0)
Overall assessment			
Normal, *n* (%)	3 (100)	3 (100)	6 (100)

Table 3. Pharmacokinetic parameters for trastuzumab by treatment dose level.

Parameter	T-DM1 3.6 mg/kg plus NPLD 45 mg/m² (*n* = 3)	T-DM1 3.6 mg/kg plus NPLD 50 mg/m² (*n* = 3)	T-DM1 3.6 mg/kg plus NPLD 60 mg/m² (*n* = 3)
	Trastuzumab mean (% CV)	Trastuzumab mean (% CV)	Trastuzumab mean (% CV)
AUC_{inf} (µg x h/mL)	569[1] (26.5)	1290[2] (NA)	709 (23.2)
AUC_{last} (µg x h/mL)	612 (26.3)	1000 (7.9)	625 (19.2)
C_{max} (µg/mL)	94.6 (16.8)	114 (2.8)	78.3 (7.6)
T_{max} (h) [a]	2.00 (1.95–25.2)	1.83 (1.83–2.02)	1.95 (1.80–2.08)
$T_{1/2}$ (days)	5.03 [1] (45.6)	11.2 [2] (NA)	7.03 (19.3)

[a] Median (minimum, maximum) are reported for T_{max}. [1] N = 2. [2] N = 1.

Table 4. Pharmacokinetic parameters for DM1 by treatment dose level.

Parameter	T-DM1 3.6 mg/kg plus NPLD 45 mg/m² (*n* = 3)	T-DM1 3.6 mg/kg plus NPLD 50 mg/m² (*n* = 3)	T-DM1 3.6 mg/kg plus NPLD 60 mg/m² (*n* = 3)
	DM1 mean (% CV)	DM1 mean (% CV)	DM1 mean (% CV)
AUC_{last} (μg x h/mL)	23.1 (143.1)	10.1 (25.0)	5.63 (24.9)
C_{max} (μg/mL)	3.76 (18.2)	8.03 (67.3)	5.13 (59.1)
T_{max} (h) [a]	2.00 (1.95–481)	1.83 (1.83–2.02)	1.95 (1.80–2.08)

[a] Median (minimum, maximum) are reported for T_{max}.

Table 5. Pharmacokinetic parameters for doxorubicinol by treatment dose level.

Parameter	T-DM1 3.6 mg/kg plus NPLD 45 mg/m² (*n* = 3)	T-DM1 3.6 mg/kg plus NPLD 50 mg/m² (*n* = 3)	T-DM1 3.6 mg/kg plus NPLD 60 mg/m² (*n* = 3)
	Doxorubicinol mean (% CV)	Doxorubicinol mean (% CV)	Doxorubicinol mean (% CV)
**CYCLE 1**			
AUC_{inf} (ng x h/mL)	1050 (26.4)	966 (36.4)	1360 (61.5)
AUC_{last} (ng x h/mL)	982 (28.4)	888 (27.8)	1340 (65.0)
C_{max} (ng/mL)	14.8 (8.4)	9.19 (42.1)	15.2 (70.6)
T_{max} (h) [a]	3.75 (3.58–3.75)	3.58 (3.58–3.92)	3.63 (3.50–3.83)
$T_{1/2}$ (days)	93.4 (37.9)	78.5 (6.0)	69.3 (11.7)
AUC (m/p) [b]	0.0231 (57.8)	0.00295 (50.0)	0.00511 (42.4)
C_{max} (m/p) [c]	0.819 (73.3)	0.106 (64.4)	0.140 (21.0)
**CYCLE 2**			
AUC_{inf} (ng x h/mL)	792 [1] (34.7)	907 [1] (30.4)	1210 [1] (71.7)
AUC_{last} (ng x h/mL)	899 (40.7)	763 (25.4)	1100 (50.3)
C_{max} (ng/mL)	16.0 (25.2)	10.1 (38.9)	15.6 (54.5)
T_{max} (h) [a]	4.02 (4.00–4.23)	3.58 (1.17–4.15)	3.78 (3.75–4.00)
$T_{1/2}$ (days)	51.91 (0.5)	64.01 (23.6)	49.41 (1.9)
AUC (m/p) [b]	0.0123 (63.4)	0.00276 (38.2)	0.00591 (44.7)
C_{max} (m/p) [c]	0.289 (60.5)	0.0997 (74.9)	0.153 (21.3)

[a] Median (minimum, maximum) are reported for T_{max}., [b] Metabolite ratio (based on AUC), calculated as AUC_{last} doxorubicinol/AUC_{last} doxorubicin.[c] Metabolite ratio (based on C_{max}), calculated as C_{max} doxorubicinol/C_{max} doxorubicin, [1] N = 2.

Table 6. The efficacy and safety of T-DM1–containing regimens for HER2-positive breast cancer.

Trial name [reference]	Year	Study Phase	Setting	Enrolled Patients, *n*	Treatment Arm (*n* patients)	Endpoint	Main findings
EMILIA [1]	2012	III	First/second line for MBC	991	T-DM1 (495) vs. lapatinib + capecitabine (496)	PFS	Median PFS with T-DM1 9.6 months vs. lapatinib + capecitabine 6.4 months (HR 0.65; 95% CI 0.55–0.77; P<0.001)
						OS	Median OS with T-DM1 30.9 months vs. median OS with lapatinib + capecitabine 25.1 months (HR 0.68; 95% CI 0.55–0.85; P <0.001)
						Safety	In T-DM1 arm, any AEs and grade ≥3 AEs were reported in 95.5% and 40.8%, respectively, and thrombocytopenia (12.9%) and elevated serum concentrations of AST (4.3%) and ALT (2.9%) were the most commonly reported grade 3 or 4 AEs. In lapatinib + capecitabine arm, any AEs and grade ≥3 AEs were reported in 97.7% and 57%, respectively, and diarrhea (20.7%) and palmar–plantar erythrodysesthesia (16.4%) were the most commonly reported grade 3 or 4 AEs.
TH3RESA [2]	2014	III	Second/third line for MBC	602	T-DM1 (404) vs. physician's choice treatment (198)	PFS	Median PFS for T-DM1 6.2 months vs. physician's choice 3.3 months (HR 0.528 [95% CI 0.422–0.661]; P <0.0001)
						OS	OS showed a trend favoring T-DM1 (HR 0.552 [95% CI 0.369–0.826] p=0·0034), but the prespecified O'Brien-Fleming stopping boundary (HR 0.370) was not crossed.
						Safety	In T-DM1 arm, any AEs and grade ≥3 AEs were reported in 94% and 32%, respectively, and

Trial name [reference]	Year	Study Phase	Setting	Enrolled Patients, *n*	Treatment Arm (*n* patients)	Endpoint	Main findings
							thrombocytopenia (5%) was the most commonly reported grade 3 or 4 AEs. In physician's choice arm, any AEs and grade ≥3 AEs were reported in 89% and 43%, respectively, and neutropenia (16%), febrile neutropenia (4%), and diarrhea (4%) were the most commonly reported grade 3 or 4 AEs.
WSG-ADAPT [3]	2015	II	Neoadjuvant	375	T-DM1 (119) vs. T-DM1 + ET (127) vs. trastuzumab + ET (129)	pCR rates of each T-DM1 arm (± ET)	pCR 41% for T-DM1 and 41.5% for T-DM1 + ET vs. 15.1% trastuzumab + ET (95% CI 15–37 for T-DM1 vs. trastuzumab + ET; 95% CI 16–37 for T-DM1 + ET vs. trastuzumab + ET; 95% CI -12–13 for T-DM1 + ET vs. T-DM1; P < 0.001)
						Safety	In the pooled T-DM1 arms, 7.5% patients suffered at least one grade ≥3 AE vs. 4.1% of trastuzumab + ET arm (P = 0.26). Most AEs were elevated serum concentrations of ALT and AST, but overall toxicity was favorable in all three treatment arms.
KAMILLA [4]	2016	III	Second/third line for MBC	2002	T-DM1 for CNS metastases at baseline (398) vs. no CNS metastases at baseline (1604)	PFS	Median for patients with CNS metastases 5.5 months (95% CI 5.3–5.6) vs. 7.7 months (95% CI 6.8–8.1) in patients without.
						OS	Median OS in patients with CNS metastases 18.9 months (95% CI 17.1–21.3) vs. 30.0 months (95% CI 27.6–31.2) in patients without (HR 1.68 [95% CI 1.46–1.93; P < 0.0001).

Trial name [reference]	Year	Study Phase	Setting	Enrolled Patients, *n*	Treatment Arm (*n* patients)	Endpoint	Main findings
						Safety	Any AEs and serious AEs were reported in 92.5% and 28.4% of patients with CNS metastases vs. 93.1% and 19.6% of patients without, respectively.<br>Headache (28.4%) and vomiting (20.4%) occurred in a slightly higher percentage of patients with CNS metastases and pyrexia (18.6%) occurred in a higher percentage of patients without CNS metastases. Nervous system AEs were more common in patients with CNS metastases (52.3%) vs. without (43.7%).
KRISTINE [5]	2016	III	Neoadjuvant	444	T-DM1 + pertuzumab (223) vs. docetaxel + carboplatin + trastuzumab, and pertuzumab (221)	pCR rate	pCR rate for T-DM1 + pertuzumab 44.4% vs. 55.7% for docetaxel + carboplatin + trastuzumab, and pertuzumab (95% CI -20.5–2.0; P=0.016).
						Safety	In T-DM1 + pertuzumab arm, any AEs and grade ≥3 AEs were reported in 88% and 13%, respectively, and decreased platelet count (1%), fatigue (1%), increased serum levels of ALT (1%), and hypokalemia (1%) were the most commonly reported grade 3 or 4 AEs.<br>In docetaxel + carboplatin + trastuzumab, and pertuzumab arm, any AEs and grade ≥3 AEs were reported in 99% and 64%, respectively, and neutropenia (25%), diarrhea (16%), and febrile neutropenia (16%) were the most commonly reported grade 3 or 4 AEs.

Trial name [reference]	Year	Study Phase	Setting	Enrolled Patients, *n*	Treatment Arm (*n* patients)	Endpoint	Main findings
MARIANNE [6]	2017	III	First line for MBC	1,095	T-DM1 + pertuzumab (363) or T-DM1 + placebo (367) vs. trastuzumab + taxane (365)	PFS	Median PFS for T-DM1 + pertuzumab 15.2 months vs. 14.1 months for T-DM1 vs. 13.7 months for trastuzumab + taxane (for T-DM1 vs trastuzumab + taxane HR 0.91 [97.5% CI 0.73–1.13]; P = 0.31); for T-DM1 + pertuzumab vs trastuzumab + taxane HR 0.87 [97.5% CI 0.69–1.08] P = 0.14); for T-DM1 + pertuzumab vs T-DM1 HR 0.91; [97.5% CI 0.73–1.13].
						Safety	Any AEs and grade ≥3 AEs were reported in 98.9% and 45.4% for T-DM1, 98.6% and 46.2% for T-DM1 + pertuzumab, and 98.6% and 54.1% for trastuzumab + taxane. In the T-DM1 arm, the most commonly reported grade ≥ 3 AEs were increased ASP (6.6%), thrombocytopenia (6.4%), and anemia (4.7%), and thrombocytopenia (7.9%), anemia (6.0%), and increased ALT (5.2%) in the T-DM1 + pertuzumab arm. With the exception of an increase in grade ≥ 3 diarrhea (2.5% vs. 0.3% for T-DM1), addition of pertuzumab to T-DM1 did not substantially increase the incidence of high-grade toxicity. The most commonly reported grade ≥ 3 AEs in the trastuzumab + taxane arm were neutropenia (19.8%), febrile neutropenia (6.5%), and diarrhea (4.2%).
THELMA	2020	Ib	First/Second line for MBC	15	NPLD+T-DM1 (15)	PFS	Median PFS in overall cohort 7.2 months (95% CI 4.5–9.6)
						ORR	ORR in overall cohort 40% (95% CI 16.3–67.7)
						Safety	Any AEs and grade ≥3 AEs were 100% and 60%. The most commonly reported grade ≥ 3 AEs was neutropenia (53.3%), thrombocytopenia (13.3%), and elevation of liver transaminases (13.3%).

Trial name [reference]	Year	Study Phase	Setting	Enrolled Patients, *n*	Treatment Arm (*n* patients)	Endpoint	Main findings

95% CI: 95% confidence interval; AE: Adverse event; ALT: Alanine aminotransferase; ASP: Aspartate aminotransferase; CI: Confidence interval; CNS: Central nervous system; ET: Endocrine therapy; HR: Hazard ratio; MBC: Metastatic breast cancer; pCR: Pathological complete response; PFS: Progression-free survival; OS: Overall survival; T-DM1: Trastuzumab emtansine.

Table 7. The efficacy of Non-Pegylated Liposomal Doxorubicin-based regimens for metastatic breast cancer.

Author	Year	Study Phase	Lines	Enrolled Patients, *n*	Treatment Arm (*n*, patients)	Endpoint	Main findings
Chan S. *et al.* [7]	2004	III	First line	160	NPLD+C (80) vs. E+C (80)	OS	Median OS for NPLD+C 18.3 months (95% CI 14.9–23.8) vs. 16.0 months (95% CI 12.8–18.3) for E+C (HR 1.55 (95% CI 0.8–1.7) P=0.504 *)
						PFS	Median PFS for NPLD+C 7.7 months (95% CI 5.4–8.9) vs. 5.6 months (95% CI 4.4–6.4) for E+C (HR 1.52 [95% CI 1.1–2.2])
						ORR	ORR for NPLD+C 46% (95.5% CI 35–58) vs. 39% (95.5% CI 28–50) for E+C (P=0.42)
Batist G. *et al.* [8]	2006	III	First line	68	NPLD (32) vs. D (36)	OS	Median OS for NPLD 16 months vs. 15 months (HR 1.12 [95.5% CI 0.63-1.98] P=0.71 *)
						PFS	Median PFS for NPLD 4.5 months vs. 3.4 months for D (HR 1.14 [95.5% CI 0.6–2.0)] P=0.66 *))
						ORR	ORR for NPLD 31% (95.5% CI 16–50) vs. 11% (95.5% CI 3–26) for D (OR 4.0 [95.5% CI 1.1–15] P=0.04 #)
Baselga J. *et al.* [9]	2014	III	First line	364	NPLD+T+P (181) vs. T+P (182)	OS	Median OS for NPLD+T+P 33.6 months (95% CI 27–38.3) vs. 29 months (95% CI 25–34.2) (HR 0.79 [95.5% 0.6–1.0] P=0.083 *)
						PFS	Median PFS for NPLD+T+P 16.1 months (95% CI 13.5–19.8) vs. 14.5 months (95% CI 12.5–16.6) (HR 0.84 [95% CI 0.6–1.1] P=0.174)
						ORR	ORR for NPLD+T+P 67% (95.5% CI 59–74) vs. 62% (95.5% CI 55–69) (HR [95% CI0.8–1.9] P=0.381)
THELMA trial	2020	Ib	First/Second line for MBC	15	NPLD+T-DM1 (15)	PFS	Median PFS for overall cohort 7.2 months (95% CI 4.5–9.6)
						ORR	ORR for overall cohort 40% (95% CI 16.3–67.7)

* P-value by log rank test. # P-value by Cochran– Mantel–Haenszel test.

95% CI: 95% confidence interval; C: Capecitabine; D: Doxorubicin; E: Epirubicin; HR: Hazard ratio; NPLD: Non-pegylated liposomal doxorubicin; OR: Odds ratio; ORR: Overall response rate; OS: Overall survival; P: Paclitaxel; PFS: Progression-free survival; T: Trastuzumab.

Table 8. The safety of Non-Pegylated Liposomal Doxorubicin-based regimens for metastatic breast cancer.

Author	Treatment Arm (*n*, patients)	Alopecia (%)	Nausea (%)	Vomiting (%)	Fatigue (%)	PPE (%)	Stomatitis (%)	Hematologic toxicity		
								TCP (%)	Anemia (%)	NTP (%)
Chan S. *et al.* [7]	NPLD+C (80)	87	21*	NR	0	0	7	4	25	87
	E+C (80)	85	19*	NR	1	0	0	3	14	67
Batist G. *et al.* [8]	NPLD (32)	NR	NR	NR	NR	0	NR	NR	NR	60
	D (36)	NR	NR	NR	NR	0	NR	NR	NR	60
Baselga J. *et al.* [9]	NPLD+T+P (181)	68	41	29	22	NR	33	NR	NR	11
	T+P (182)	63	23	13	15	NR	13	NR	NR	1
THELMA trial	NPLD+T-DM1 (15)	26.7	60	13.3	13.3	NR	NR	13.3	26.7	53.3

* Total number of nausea/vomiting.

C: Capecitabine; D: Doxorubicin; E: Epirubicin; NPLD: Non-pegylated liposomal doxorubicin; NR: Not reported; NTP: Neutropenia; P: Paclitaxel; PPE: Palmar-plantar erythrodysesthesia; T: Trastuzumab; T-DM1: Trastuzumab emtansine; TCP: Thrombocytopenia.

References.

1. Verma, S.; Miles, D.; Gianni, L.; Krop, I.E.; Welslau, M.; Baselga, J.; Pegram, M.; Oh, D.-Y.; Diéras, V.; Guardino, E.; et al. Trastuzumab emtansine for HER2-positive advanced breast cancer. *N. Engl. J. Med.* **2012**, *367*, 1783–1791, doi:10.1056/NEJMoa1209124.
2. Krop, I.E.; Kim, S.-B.; González-Martín, A.; LoRusso, P.M.; Ferrero, J.-M.; Smitt, M.; Yu, R.; Leung, A.C.F.; Wildiers, H.; TH3RESA study collaborators Trastuzumab emtansine versus treatment of physician's choice for pretreated HER2-positive advanced breast cancer (TH3RESA): A randomised, open-label, phase 3 trial. *Lancet Oncol.* **2014**, *15*, 689–699, doi:10.1016/S1470-2045(14)70178-0.
3. Harbeck, N.; Gluz, O.; Christgen, M.; Kates, R.E.; Braun, M.; Küemmel, S.; Schumacher, C.; Potenberg, J.; Kraemer, S.; Kleine-Tebbe, A.; et al. De-Escalation Strategies in Human Epidermal Growth Factor Receptor 2 (HER2)–Positive Early Breast Cancer (BC): Final Analysis of the West German Study Group Adjuvant Dynamic Marker-Adjusted Personalized Therapy Trial Optimizing Risk Assessment and Therapy Response Prediction in Early BC HER2- and Hormone Receptor–Positive Phase II Randomized Trial—Efficacy, Safety, and Predictive Markers for 12 Weeks of Neoadjuvant Trastuzumab Emtansine With or Without Endocrine Therapy (ET) Versus Trastuzumab Plus ET. *JCO* **2017**, *35*, 3046–3054, doi:10.1200/JCO.2016.71.9815.
4. Montemurro, F.; Delaloge, S.; Barrios, C.H.; Wuerstlein, R.; Anton, A.; Brain, E.; Hatschek, T.; Kelly, C.M.; Peña-Murillo, C.; Yilmaz, M.; et al. Trastuzumab emtansine (T-DM1) in patients with HER2-positive metastatic breast cancer and brain metastases: Exploratory final analysis of cohort 1 from KAMILLA, a single-arm phase IIIb clinical trial☆. *Annals of Oncology* **2020**, *31*, 1350–1358, doi:10.1016/j.annonc.2020.06.020.
5. Hurvitz, S.A.; Martin, M.; Symmans, W.F.; Jung, K.H.; Huang, C.-S.; Thompson, A.M.; Harbeck, N.; Valero, V.; Stroyakovskiy, D.; Wildiers, H.; et al. Neoadjuvant trastuzumab, pertuzumab, and chemotherapy versus trastuzumab emtansine plus pertuzumab in patients with HER2-positive breast cancer (KRISTINE): A randomised, open-label, multicentre, phase 3 trial. *Lancet Oncol* **2018**, *19*, 115–126, doi:10.1016/S1470-2045(17)30716-7.
6. Perez, E.A.; Barrios, C.; Eiermann, W.; Toi, M.; Im, Y.-H.; Conte, P.; Martin, M.; Pienkowski, T.; Pivot, X.; Burris, H.A.; et al. Trastuzumab Emtansine With or Without Pertuzumab Versus Trastuzumab Plus Taxane for Human Epidermal Growth Factor Receptor 2–Positive, Advanced Breast Cancer: Primary Results From the Phase III MARIANNE Study. *J Clin Oncol* **2017**, *35*, 141–148, doi:10.1200/JCO.2016.67.4887.
7. Chan, S.; Davidson, N.; Juozaityte, E.; Erdkamp, F.; Pluzanska, A.; Azarnia, N.; Lee, L.W. Phase III trial of liposomal doxorubicin and cyclophosphamide compared with epirubicin and cyclophosphamide as first-line therapy for metastatic breast cancer. *Ann. Oncol.* **2004**, *15*, 1527–1534, doi:10.1093/annonc/mdh393.
8. Batist, G.; Harris, L.; Azarnia, N.; Lee, L.W.; Daza-Ramirez, P. Improved anti-tumor response rate with decreased cardiotoxicity of non-pegylated liposomal doxorubicin compared with conventional doxorubicin in first-line treatment of metastatic breast cancer in patients who had received prior adjuvant doxorubicin: Results of a retrospective analysis. *Anticancer Drugs* **2006**, *17*, 587–595, doi:10.1097/00001813-200606000-00014.
9. Baselga, J.; Manikhas, A.; Cortés, J.; Llombart, A.; Roman, L.; Semiglazov, V.F.; Byakhov, M.; Lokanatha, D.; Forenza, S.; Goldfarb, R.H.; et al. Phase III trial of nonpegylated liposomal doxorubicin in combination with trastuzumab and paclitaxel in HER2-positive metastatic breast cancer. *Annals of Oncology* **2014**, *25*, 592–598, doi:10.1093/annonc/mdt543.

Publisher's Note: MDPI stays neutral with regard to jurisdictional claims in published maps and institutional affiliations.

© 2020 by the authors. Licensee MDPI, Basel, Switzerland. This article is an open access article distributed under the terms and conditions of the Creative Commons Attribution (CC BY) license (http://creativecommons.org/licenses/by/4.0/).
